# Nuclear Receptors in Bladder Cancer: Insights into miRNA-Mediated Regulation and Potential Therapeutic Implications

**DOI:** 10.3390/ijms26157340

**Published:** 2025-07-29

**Authors:** José Javier Flores-Estrada, Adriana Jiménez, Georgina Victoria-Acosta, Enoc Mariano Cortés-Malagón, María Guadalupe Ortiz-López, María Elizbeth Alvarez-Sánchez, Stephanie I. Nuñez-Olvera, Yussel Fernando Pérez-Navarro, Marcos Morales-Reyna, Jonathan Puente-Rivera

**Affiliations:** 1 División de Investigación, Hospital Juárez De México, Mexico City 07760, Mexico; javier_70_1999@yahoo.com (J.J.F.-E.); adijh@hotmail.com (A.J.); georgina.acostaa@salud.gob.mx (G.V.-A.); emcortes@cinvestav.mx (E.M.C.-M.); gortizl@prodigy.net.mx (M.G.O.-L.); 2 Genetics Laboratory, Hospital Nacional Homeopático, Mexico City 06800, Mexico; 3 Laboratorio de Patogénesis Celular Humana y Veterinaria Posgrado en Ciencias Genómicas, Universidad Autónoma de la Ciudad de México (UACM), San Lorenzo 290, Col. Del Valle, Mexico City 03100, Mexico; maria.alvarez@uacm.edu.mx (M.E.A.-S.); yussel.perez@estudiante.uacm.edu.mx (Y.F.P.-N.); marcos.morales.reyna@alumnos.uacm.edu.mx (M.M.-R.); 4 Departamento de Biología Celular y Fisiología, Instituto de Investigaciones Biomédicas, Universidad Nacional Autónoma de México, Mexico City 04510, Mexico; iraiz.nunez@iibiomedicas.unam.mx

**Keywords:** bladder cancer, nuclear receptors, miRNAs, therapeutic response, gene regulation, RNA-based therapy

## Abstract

Nuclear receptors (NRs) are ligand-activated transcription factors that regulate gene expression and are involved in diverse physiological and pathological processes, including carcinogenesis. In bladder cancer (BCa), dysregulation of NR signaling pathways has been linked to tumor initiation, progression, therapy resistance, and immune evasion. Recent evidence highlights the intricate crosstalk between NRs and microRNAs (miRNAs), which are small non-coding RNAs that posttranscriptionally modulate gene expression. This review provides an integrated overview of the molecular interactions between key NRs and miRNAs in BCa. We investigated how miRNAs regulate NR expression and function and, conversely, how NRs influence miRNA biogenesis, thereby forming regulatory feedback loops that shape tumor behavior. Specific miRNA–NR interactions affecting epithelial-to-mesenchymal transition, metabolic reprogramming, angiogenesis, and chemoresistance are discussed in detail. Additionally, we highlight therapeutic strategies targeting NR–miRNA networks, including selective NR modulators, miRNA mimics and inhibitors, as well as RNA-based combinatorial approaches focusing on their utility as diagnostic biomarkers and personalized treatment targets. Understanding the molecular complexity of NR–miRNA regulation in BCa may open new avenues for improving therapeutic outcomes and advancing precision oncology in urological cancers.

## 1. Introduction

Bladder cancer (BCa) is the tenth most common malignancy worldwide, with more than 573,000 new cases and 212,000 deaths reported in 2020 alone [1,2,3]. It remains a major public health burden due to its high recurrence rate, treatment resistance, and limited improvement in survival over the past decades [4]. Despite advances in surgical techniques, chemotherapy, and immunotherapy, clinical outcomes for advanced or recurrent BCa remain poor [5,6].

BCa is characterized by high molecular and clinical heterogeneity, encompassing nonmuscle-invasive (NMIBCa) and muscle-invasive BCa (MIBCa) subtypes with distinct genetic landscapes and progression patterns [7,8,9]. Genetic alterations in pathways involving TP53, FGFR3, and PIK3CA, and chromatin remodeling are frequently observed in BCa and contribute to therapeutic resistance [10,11,12]. In addition to somatic mutations, epigenetic changes and transcriptional dysregulation have emerged as key drivers of urothelial carcinogenesis [13,14].

Nuclear receptors (NRs), a superfamily of ligand-activated transcription factors, regulate genes involved in cell proliferation, differentiation, metabolism, and the immune response [15,16]. NRs such as androgen receptor (AR), estrogen receptors (ERα and ERβ), peroxisome proliferator-activated receptors (PPARs), and glucocorticoid receptor (GCR) are implicated in BCa pathogenesis through their modulation of oncogenic and tumor suppressive pathways [17,18,19,20,21,22]. The expression and activity of NRs vary among BCa subtypes and are associated with patient prognosis and therapeutic response [23,24].

Importantly, microRNAs (miRNAs), short, non-coding RNAs that regulate gene expression posttranscriptionally, directly target NRs or modulate their downstream signaling networks. Dysregulated miRNAs can function as oncogenes or tumor suppressors in BCa, influencing proliferation, apoptosis, angiogenesis, immune evasion, and metastasis [25,26,27]. Moreover, NRs also regulate miRNA biogenesis, creating feedback loops that fine-tune transcriptional programs during cancer progression [28,29,30].

Recent evidence highlights the therapeutic potential of targeting NR–miRNA axes via selective NR modulators, miRNA mimics or inhibitors, and combination approaches that increase sensitivity to chemotherapy or immunotherapy. Understanding these interactions is crucial to the development of precision medicine in BCa, particularly in the context of treatment-resistant or high-grade tumors [31,32,33,34].

In this review, we summarize the molecular functions of key NRs in BCa, their regulation by miRNAs, and the therapeutic implications of targeting these networks. We also highlight emerging RNA-based interventions and combinatorial strategies that may improve clinical outcomes for BCa patients.

## 2. Nuclear Receptors and miRNAs as Key Regulators in BCa

**Figure 1:**
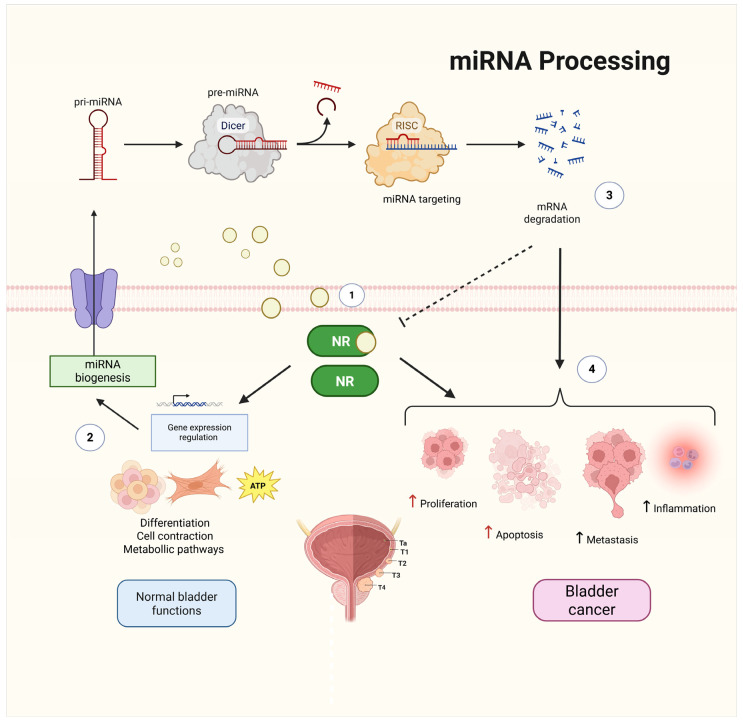
Interplay between NRs and miRNAs in bladder homeostasis and cancer. NRs regulate gene expression by binding to specific ligands and modulating transcriptional programs involved in cell differentiation, contraction, and metabolism, contributing to normal bladder functions (1,2). miRNAs, which are synthesized through canonical biogenesis pathways involving Dicer and the RNA-induced silencing complex (RISC), fine-tune gene expression posttranscriptionally (3). Cross-regulation occurs as NRs modulate miRNA biogenesis and transcription, whereas miRNAs regulate NR expression, establishing feedback loops. Dysregulation of these interactions contributes to BCa pathophysiology by increasing proliferation, metastasis, inflammation, and evasion of apoptosis (4). Solid black straight arrows indicate direct sequential processes, red and black up arrows indicate increased activity, while dashed arrow represents inhibition process. Created with BioRender.com (accessed on 1 May 2025).

NRs are ligand-activated transcription factors that regulate gene expression in response to steroid hormones, retinoids, fatty acids, and other lipophilic molecules [35]. By binding to specific DNA response elements, NRs control the transcription of genes involved in metabolism, immune modulation, development, and cell fate determination [36,37]. In cancer, NRs often exhibit altered expression, somatic mutations, or epigenetic deregulation that disrupt normal transcriptional programs and contribute to oncogenesis [38,39] (Figure 1).

Emerging evidence has demonstrated that NRs function within complex regulatory pathways that involve miRNAs [40,41,42]. miRNAs are small (~22 nt), non-coding RNAs that posttranscriptionally repress gene expression by targeting the 3′ untranslated region (3′-UTR) of mRNAs, leading to mRNA degradation or translational inhibition [43,44]. More than 60% of human protein-coding genes are under miRNA control, and their dysregulation is a hallmark of multiple cancers that act as either tumor suppressors or oncogenes (oncomiRs) depending on the molecular context [12,35].

As oncomiRs, these ncRNAs enhance cell proliferation, survival, and therapy resistance, promoting tumor growth [35]. These oncomiRs are also capable of modulating NR signaling pathways, reinforcing oncogenic programs [45,46]. As tumor suppressors, miRNAs inhibit tumor growth by promoting apoptosis, modulating chemoresistance, suppressing BCa cell adhesion, progression, and immune evasion [36,37,40,41,42,43], or exerting antiangiogenic and antimetastatic effects [40].

The role of specific miRNAs in BCa appears to be complex. While they are over-expressed, leading to the reduction of pathways involved in cell proliferation, they also target key regulators of the cell cycle and are influenced by lncRNAs that negatively regulate their function. These lncRNAs promote resistance to apoptosis, migration, invasion, and cancer stemness, further complicating the therapeutic targeting of miRNAs in BCa [46,47,48,49]. These examples illustrate how miRNAs participate in complex regulatory circuits with NRs and their downstream effectors, acting either to constrain or promote tumor development depending on the signaling milieu. Table 1 and Table 2 summarize key examples of oncogmiRs and tumor-suppressive miRNAs, respectively, involved in BCa.

NR–miRNA crosstalk can occur in three main directions: (1) miRNAs directly target NR transcripts, modulating their expression and activity; (2) NRs transcriptionally regulate miRNA genes, often via hormone response elements (HREs); and (3) NRs influence miRNA processing, including Drosha- and Dicer-mediated cleavage of primary miRNAs [47,48,49,50,51]. These feedback and feedforward loops fine-tune processes critical to cancer progression, such as epithelial-to-mesenchymal transition (EMT), proliferation, apoptosis, angiogenesis, and immune evasion [52,53,54,55] or even promote chemoresistance and proliferation [56,57].

Although such NR–miRNA interactions have been extensively characterized in hormone-driven malignancies like breast and prostate cancer (PCa) [58,59,60], their roles in BCa have only recently begun to emerge [61,62,63,64]. However, the clinical and mechanistic significance of many of these interactions remains poorly understood.

In the following sections, we analyze the prominent NR families involved in BCa—androgen receptor (AR), estrogen receptor (ER), peroxisome proliferator-activated receptor (PPAR), glucocorticoid receptor (GCR), and orphan nuclear receptors (ONRs)—focusing on their miRNA-mediated regulation, downstream signaling consequences, and potential for therapeutic targeting.

**Table 1:** OncomiRs involved in BCa.

miRNA (Expression)	Target/Regulation	Effect on BCa	References
miR-23a, miR-141b, miR-205 (↑)	Repress ZEB2, PTEN, and E-cadherin, respectively	Promote tumor invasion and progression	[31]
miR-525-5p, miR-144 (↑)	Repress SLPI; upregulate GRβ (Glucocorticoid Receptor β)	Promote metástasis	[52,53]
miR-92a, miR-19a, miR-130 (↑)	Repress DAB2IP, PTEN, RUNX3	Promote tumor progression	[31,38]
miR-21, miR-221/222, miR-125b, miR-133b (↑)	Repress PTEN, PDCD4, p27Kip1, p57, E2F3; downregulate p-PI3K, p-AKT; upregulate AKT1	Promote tumor growth and proliferation	[44,46,54]
miR-193a-3p (↑)	Repress Homeobox C9 (HOXC9) gene	Promote multidrug resistance	[55]
miR-29c (↓)	Regulates CDK6	Regulate cell growth and invasion in vitro	[56]

(↑) Overexpressed. (↓) Downexpressed.

**Table 2:** Tumor suppressor miRNAs involved in BCa.

miRNA (Expression)	Target/Regulation	Effect on BCa	References
miR-34a, miR-145 (↓)	Repress CD44, PD-L1	Decrease adhesion, invasion, and immune evasion	[36,40,41]
miR-125a, miR-125b (↓)	Targets HK2 suppressing PI3K-Akt, targets FUT4	Decreases migration, invasion, progression, and modulates apoptosis	[46,57]
miR-449a (↑)	Promotes AR degradation, targets CDK6 and CDC25a, and activates accumulation of the pocket proteins Rb and p130	Decreases cell proliferation	[58,59]
miR-490-5p, miR-139 (↓)	Represses EGFR and MMP11expression	Reduces invasiveness	[60,61,65]
miR-101 (↓)	Target genes regulating the cell cycle	Decreases proliferation	[50]
miR-203a (↓)	Targets SIX4	Decreases tumor growth	[51]

(↑) Overexpressed. (↓) Downexpressed.

## 3. Androgen Receptor Signaling and Its Regulation by Non-Coding RNAs in BCa

AR is a ligand-dependent transcription factor belonging to the NR superfamily. It is activated by endogenous androgens, such as testosterone and dihydrotestosterone [62]. Structurally, AR consists of four major functional domains: the N-terminal transactivation domain (NTD), the DNA-binding domain (DBD), the hinge region, and the ligand-binding domain (LBD) [63]. Upon ligand binding, AR undergoes conformational changes, allowing it to bind to androgen response elements (AREs) and activate transcription of target genes involved in proliferation, differentiation, and survival [64,66]. AR is widely expressed across human tissues, playing critical roles in various physiological processes. In the male reproductive system, AR is abundant in prostatic epithelial cells, Leydig cells, and Sertoli cells, where it regulates spermatogenesis and hormone secretion. In females, AR expression is found in ovarian thecal and granulosa cells, the uterus, and mammary glands, influencing reproductive and endocrine functions [67,68]. Beyond reproductive organs, AR is involved in systemic processes, including immunomodulation, inflammation, and autoimmunity [69].

In the context of BCa, AR is expressed in basal and luminal urothelial cells, stromal fibroblasts, and smooth muscle cells, with higher expression levels observed in males. This sex-specific expression pattern correlates with the increased incidence of BCa in men [70]. Experimental evidence suggests that AR signaling supports urothelial homeostasis, but when dysregulated, it can promote carcinogenesis. For example, AR depletion in mouse models reduces susceptibility to chemically induced bladder tumors, highlighting the protumorigenic role of AR [71,72]. Upon activation, AR enhances cell proliferation, migration, and survival through upregulation of oncogenes such as CD24 and EGFR, and activation of key signaling pathways like PI3K/AKT, MAPK, and Wnt/β-catenin. Clinically, AR expression serves as a prognostic marker, particularly in NMIBCa, where it is associated with increased recurrence risk. Current clinical trials are evaluating therapies that target AR, including androgen deprivation therapy (ADT) and AR antagonists such as enzalutamide and bicalutamide [73,74,75,76,77].

In addition to androgen signaling, ncRNAs, especially miRNAs and long noncoding RNAs (lncRNAs), play crucial roles in the fine-tuned regulation of AR signaling in BCa. These ncRNAs exert their effects through mechanisms such as mRNA degradation or translational repression, thereby modulating AR activity. One well-characterized regulatory axis involves miR-124, XIST, and AR. In BCa cell lines such as T24 and 5637, miR-124 functions as a tumor suppressor by directly targeting AR mRNA, thereby inhibiting androgen-induced proliferation and migration. The lncRNA XIST, which is overexpressed in BCa tissues and cell models, acts as a competing endogenous RNA (ceRNA), sequestering miR-124 and restoring AR expression, thereby increasing tumor aggressiveness. Functional studies have demonstrated that knockdown of XIST or restoration of miR-124 reduces cell migration and proliferation, suggesting that this axis is a potential therapeutic target [78,79,80].

Another significant regulatory loop involves miR-21, a well-established oncomiR frequently overexpressed in patient-derived BCa tissues and cell lines. miR-21 inhibits PTEN, leading to activation of the PI3K/AKT/mTOR pathway and indirectly enhancing AR transcriptional activity. Notably, AR itself upregulates miR-21, creating a positive feedback loop that further amplifies oncogenic signaling. In vivo studies using ADM-21, a miR-21 inhibitor, have shown reduced tumor growth and PTEN restoration in xenograft models, underscoring the therapeutic potential of targeting the miR-21/PTEN/AR axis [81]. Furthermore, the miR-525-5p/SLPI regulatory complex has context-specific effects. In BCa cell lines, AR activation induces miR-525-5p, which suppresses SLPI, a vasculogenic mimicry (VM) inhibitor, thereby facilitating metastatic potential. In contrast, in PCa cell lines, AR represses miR-525-5p, preserving SLPI expression and suppressing VM. This finding illustrates how the role of AR can vary significantly between tissue types [52]. These context-specific effects are also supported by pharmacologic inhibition of AR via bicalutamide, which enhances invasiveness in PCa cells through the circRNA-ARC1/miR-125b-2-3p axis but suppresses BCa invasion through the miR-4736/PPARγ/MMP-9 axis, suggesting the importance of tailoring anti-AR therapies on the basis of tumor type [74].

Several other AR-regulating miRNAs have shown therapeutic potential. For example, miR-449a, which is downregulated in advanced BCa tissues and cell lines, directly targets AR mRNA, suppressing its downstream gene programs and enhancing chemosensitivity [58,82]. Similarly, miR-200a-3p acts as a tumor suppressor by downregulating STAT4 and modulating PD-L1 expression through its interaction with AR-induced circRNAs. Notably, miR-200a-3p is downregulated in muscle-invasive BCa but detectable in patient serum, suggesting its potential as a diagnostic or prognostic biomarker [83,84,85].

**Figure 2:**
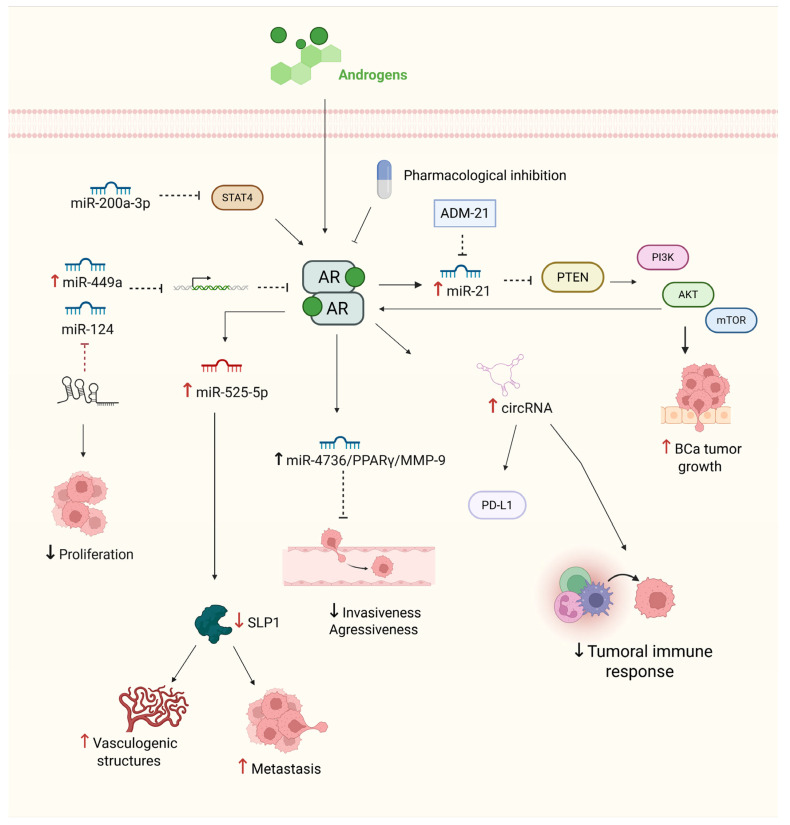
Androgen receptor (AR) signaling network and its regulation by ncRNAs in BCa. The figure illustrates key molecular interactions involved in AR signaling and its downstream effects in BCa. Androgen binding activates AR, promoting tumor progression through the PI3K/AKT/mTOR axis, immune evasion, and tumor growth. AR also modulates the expression of various ncRNAs, including miR-21, miR-525-5p, miR-4736, and circRNAs, which contribute to increased proliferation, invasion, and metastasis. Conversely, tumor-suppressive miRNAs, such as miR-124, miR-449a, and miR-200a-3p, negatively regulate AR or its downstream targets, thereby limiting proliferation and vascular mimicry. Pharmacological inhibition, the use of miRNA inhibitors (ADM-21), and AR-miRNA crosstalk represent potential therapeutic strategies to modulate aggressiveness and immune escape in BCa. Solid straight black arrows indicate activation, red and black up arrows indicate increased expression, red and black down arrows indicate repressed activity, dashed red and black arrows represent inhibition process. Created with BioRender.com (accessed on 1 May 2025).

Taken together, these findings underscore AR as a central player in BCa biology, with its activity intricately regulated by a complex network of ncRNAs. From mechanistic studies in cell lines and animal models to biomarker validation in patient cohorts, evidence supports the idea that AR–ncRNA axes govern tumor growth, invasion, immune evasion, and treatment response. Understanding these multilayered interactions is essential for the development of RNA-based therapeutics and personalized AR-targeted interventions (Figure 2).

## 4. Molecular Roles of Estrogen Receptors in BCa: From Transcriptional Control to miRNA-Driven Networks

Two subtypes of ERs, ERα and ERβ, mediate the effects of estrogens by regulating gene expression in response to hormone binding. Upon estrogen binding, ERs undergo a conformational change, translocate to the nucleus, and bind to estrogen response elements (EREs) in the promoter regions of target genes involved in cell proliferation, differentiation, and survival [86,87]. While both ER subtypes are expressed in BCa, they play distinct and sometimes opposing roles in cancer progression. In hormone-dependent cancers such as breast cancer, ERα is generally associated with promoting cell proliferation, whereas ERβ has been linked to inhibitory effects on cell growth and migration. However, in BCa, ERα is considered to have a tumor-suppressive and protective role; instead, ERβ has an oncogenic effect [88,89].

As mentioned above, ERα and ERβ have context-dependent effects on BCa, with ERα often playing a protective role and ERβ acting as an oncogene in more aggressive subtypes. For example, ERβ has been shown to regulate minichromosome maintenance complex component 5 (MCM5), a key regulator of DNA replication and cell proliferation, suggesting that ERβ activation contributes to tumor progression. Furthermore, the proliferative and invasive effects of ERβ have been demonstrated in BCa cell lines (UMUC3 and J82), where ERβ signaling also enhances cisplatin resistance by increasing β-catenin activity and promoting tumor growth [38,89,90].

Conversely, ERα has tumor-suppressive functions in BCa, primarily by inhibiting AKT signaling. This phenomenon has been demonstrated in ERα-knockout mice, which exhibit an increased incidence of cancer. Furthermore, ERα activation induces the expression of INPP4B, an enzyme that limits AKT activity and thereby prevents tumor progression [91,92].

Notably, estrogens are implicated in advanced-stage BCa and have been linked to worse survival outcomes, particularly in females. Decreased levels of biologically active estrogens could lead to increased tumor proliferation and progression, highlighting the need for therapeutic strategies targeting estrogen signaling. Selective estrogen receptor modulators (SERMs) have been shown to suppress BCa cell proliferation. SERMs act as agonists or antagonists in a tissue-specific manner and can exert their antiproliferative effects on BCa by activating the ER [93], probably ERα, or by inhibiting ERβ, as observed in BCa cell lines treated with raloxifene [94].

The relationship between ERs and miRNAs in BCa further complicates their functional roles. miR-92a, a well-established oncogenic miRNA, is upregulated in ERβ-positive BCa cell lines. This miRNA is induced by ERβ binding to its host gene C13orf25 and induces cell proliferation and invasion by downregulating the tumor suppressor DAB2IP [38]. In contrast, miR-490-5p suppresses EGFR expression and is induced by ERα through the inhibition of circ_0023642, which acts as a sponge for miR-490-5p. This sponge function results in reduced invasiveness in BCa cells, suggesting that ERα-induced miR-490-5p expression may be a protective mechanism against tumor metastasis [61].

Additionally, miR-4324, another miRNA regulated by ERα, reduces cell proliferation and metastasis by targeting RACGAP1. The overexpression of miR-4324 in BCa cells enhances doxorubicin sensitivity, providing a potential therapeutic avenue for ERα-positive BCa tumors [95]. Furthermore, miR-206, which is downregulated in BCa, suppresses YRDC expression, reducing cell proliferation, migration, and colony formation. The restoration of miR-206 in BCa cells resulted in a significant reduction in tumorigenic potential in vitro and in vivo [96].

The miR-221/222 cluster is another miRNA family linked to BCa progression. Although not directly regulated by ERα, miR-221/222 promotes EMT and tumor invasiveness in BCa by targeting p53 and PUMA [97]. These miRNAs also exhibit therapeutic importance, as their inhibition reduces metastasis and improves chemosensitivity in BCa models.

The complex regulatory relationship between ERα, ERβ, and their downstream miRNAs in BCa highlights the necessity of precise molecular profiling when therapeutic strategies targeting estrogen signaling are designed. Both ERα and ERβ contribute significantly to BCa progression, and targeting their pathways may offer novel strategies for treatment, particularly in ER-positive tumors.

**Figure 3:**
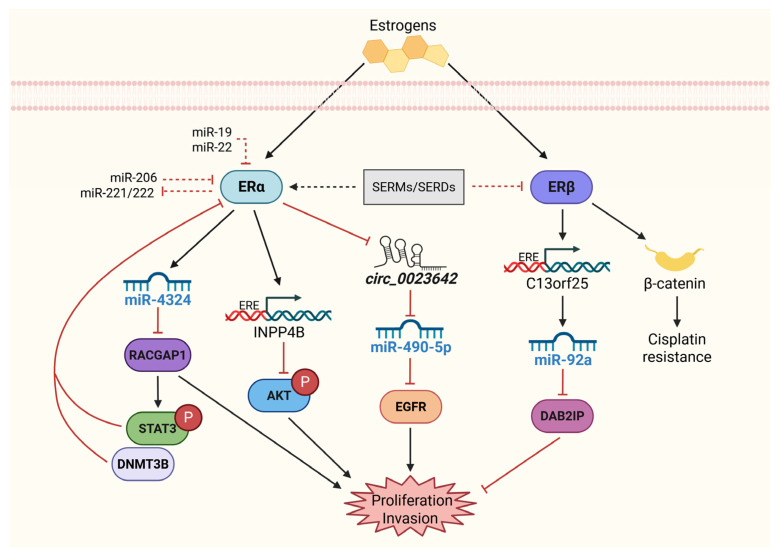
Role of the ER in the proliferation and invasion of BCa. In contrast to other cancer types, ERα and ERβ have anti- and pro-oncogenic effects on BCa. ERα can reduce cell proliferation and invasion by inhibiting EGFR, AKT, and RACGAP through regulatory mechanisms that could involve miRNA participation. In turn, ERα can be repressed by STAT3 and DNMT3B, which facilitates its methylation. In addition, miR-19, miR-22, miR-206, and miR-221/222 downregulated ERα. On the other hand, ERβ increases cell proliferation and invasion by indirectly inhibiting DAB2IP and promoting cisplatin resistance via β-catenin overexpression. Selective estrogen receptor modulators (SERMs) and selective estrogen receptor degraders (SERDs) may inhibit ER signaling in BCa since decreased levels of biologically active estrogens could lead to increased BCa malignancy. Solid black straight arrows indicate activation, dashed red arrows represent indirect inhibition and solid red arrows indicate direct inhibition process. Created with BioRender.com (accessed on 1 May 2025).

Figure 3 provides an overview of the ER–miRNA networks involved in regulating BCa progression, illustrating the functional divergence between ERα and ERβ signaling and their potential therapeutic implications.

## 5. Molecular Functions of Orphan Nuclear Receptors in BCa: miRNA Regulatory Networks and Cancer Development and Progression

ONRs, a subclass of NRs that lack known endogenous ligands, are critical regulators of gene expression in various biological processes, including development, cell differentiation, and metabolic homeostasis. These receptors have gained considerable attention for their involvement in the biology of tumors, including BCa. For example, retinoic acid-related orphan receptor C (RORC) has been linked to cell proliferation and the regulation of immune responses, making it a potential target for cancer therapy [98,99] because its expression can be associated with both tumor progression and regression, depending on the tumor subtype [100].

In the clinical context, targeting RORC in BCa has the potential to modulate immune responses and inhibit tumor growth, but its variable expression in tumor subtypes complicates its universal therapeutic application. Thus, while RORC is a promising therapeutic target, its application must be tailored to specific BCa subtypes where its expression is prevalent, highlighting the necessity for personalized medicine strategies. Nur77 (NR4A1) and HNF4α (Hepatocyte Nuclear Factor 4 Alpha) further contribute to BCa biology by regulating gene expression in cancer cells. Nur77, in particular, can induce apoptosis in BCa cells, positioning it as a therapeutic target for inhibiting tumor growth [101,102]. However, its abnormal expression may also regulate tumor growth and drug sensitivity, complicating its role in BCa progression [103,104]. The dual role of Nur77 in promoting apoptosis while potentially enhancing drug resistance poses a significant challenge for the development of Nur77-targeted therapies, which may require combination treatments to balance its proapoptotic and resistance-associated functions.

RORα (RAR-related orphan receptor alpha), known for its involvement in cancer-related inflammation, also modulates the tumor response, although further research is needed to fully understand its effects in BCa [105,106]. Additionally, RORC regulates cell proliferation and chemotherapy resistance through the PD-L1/ITGB6/STAT3 signaling axis, highlighting its role in tumor progression and therapeutic resistance [99]. These findings suggest that combination therapies targeting both RORC and immune checkpoints could enhance therapeutic outcomes by addressing immune evasion and tumor growth.

On the other hand, HNF4G has been shown to promote BCa cell growth and invasion by regulating hyaluronan synthase 2, a gene associated with cell motility in cancer. Interestingly, HNF4G overexpression acts as an oncogene in BCa tissues, but its inhibition via miR-34a significantly reduces cell viability, colony formation, and invasion, suggesting its potential as a therapeutic target. miR-34a, which acts as a tumor suppressor, reduces the expression of HNF4G by binding to its 3′-UTR, promoting its degradation through the RISC pathway. This action reduces HNF4G levels and limits the activation of protumorigenic genes associated with cell proliferation and EMT, thus reducing tumor invasion and metastasis However, the oncogenic role of HNF4G needs careful evaluation in clinical trials, as its overexpression may be a marker of aggressive BCa, and treatments that can selectively target overexpressed oncogenes without affecting normal cellular functions are needed [107,108,109].

Nur77, although it has the opposite effect in BCa, is also implicated in regulating the immune response and modulating inflammation; however, specific studies supporting this claim are still lacking in BCa [110]. The targeting of miR-34a for therapeutic use is promising; however, its role as a tumor suppressor must be balanced with its potential effects on other signaling pathways, which could complicate its clinical application.

**Figure 4:**
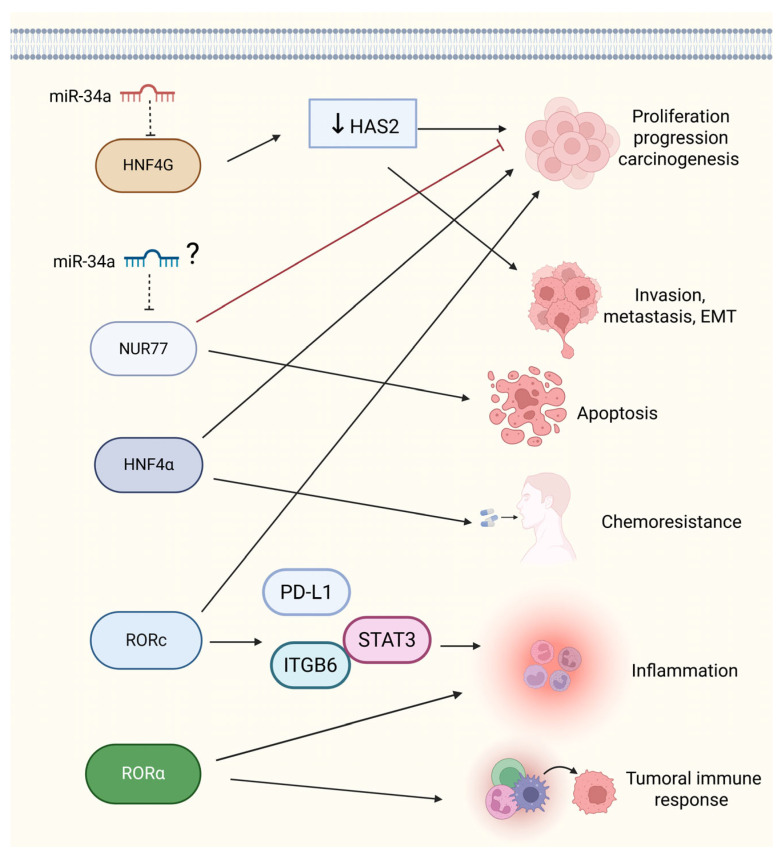
Molecular roles of ONR and its regulation by miRNAs in BCa progression. Several RNRs play pivotal roles in BCa through the modulation of tumor cell behavior and interaction with the tumor microenvironment. HNF4G, which is directly targeted by miR-34a, promotes proliferation and progression via hyaluronan synthase 2 (HAS2). Nur77 and HNF4α influence apoptosis, invasion, and chemoresistance, although the regulation of Nur77 by miR-34a in BCa remains to be clarified. RORC activates the PD-L1/ITGB6/STAT3 signaling axis, thereby enhancing inflammation and therapy resistance. Additionally, RORα may regulate tumor immune responses. These ONR-mediated pathways represent potential targets for therapeutic intervention in BCa. Question mark (?) indicates speculative not validated function, solid straight black arrows indicate activation, dashed black arrow represents indirect inhibition, black down arrow indicates repressed activity and solid red arrow indicates direct inhibition process. Figure created with BioRender.com (accessed on 1 May 2025).

In summary, ONRs such as RORC, Nur77, HNF4α, and HNF4G play pivotal and context-dependent roles in BCa development and progression. Their expression can either promote or inhibit tumor progression, depending on the receptor and cancer subtype. miRNAs, such as miR-34a, modulate these pathways, influencing critical cancer hallmarks, including proliferation, invasion, and metastasis. Notably, the increased expression of these ONRs in BCa tissues has been associated with poor patient outcomes, suggesting their use as prognostic biomarkers [111]. While targeting these molecular pathways presents exciting therapeutic opportunities, clinical limitations, including tumor subtype heterogeneity, treatment resistance, and the need for combination therapies, must be considered. Further research is needed to explore the conflicting data regarding the roles of ONR in BCa and to refine therapeutic strategies that can specifically address these challenges. Understanding the regulatory mechanisms of ONRs, particularly their interactions with miRNAs, may provide crucial insights into the development of more effective, personalized treatments for BCa patients (Figure 4).

## 6. PPARγ and the Tumor Microenvironment in BCa: Molecular Regulation

PPARs are nuclear transcription factors belonging to the nuclear hormone receptor superfamily. The PPAR subfamily comprises three subtypes, namely, PPARα, PPARβ/δ, and PPARγ, each encoded by distinct genes [112]. Among them, PPARγ has been extensively studied because of its involvement in various biological processes, such as adipocyte differentiation, lipid metabolism, insulin sensitization, and immune modulation. PPARγ acts as a key transcription factor in BCa, especially within the luminal subtype, and plays a crucial role in regulating urothelial differentiation in both mouse models and organoids in vitro [113,114]. In BCa, PPARγ is upregulated in luminal subtypes and downregulated in basal subtypes, particularly in MIBCa [113,115].

Depending on the tumor microenvironment, PPARγ activation can have both tumor-promoting and tumor-suppressive effects. PPARγ forms a heterodimer with retinoid X receptor α (RXRa), which binds DNA at peroxisome proliferator response elements (PPREs). The receptor exists in complexes with corepressor or coactivator proteins, which influence gene expression through chromatin remodeling, depending on its activation state [115,116]. In the luminal subtype, PPARγ positively controls genes associated with tumor cell differentiation, whereas in the basal subtype, high PPARγ activity is linked to tumor progression and immune evasion [117].

Mutations in RXRa (S427F/Y) and PPARγ (M280I, I290M, T475M), along with high levels of PPARγ expression at the mRNA level, are often observed in MIBCa, even in the absence of gene amplification. These alterations lead to a dependence on PPARγ for tumor progression, where the formation of the PPARγ/RXRa heterodimer activates pathways that suppress immune cell activity by altering the expression of inflammatory chemokines. This disruption of immune signaling contributes to immune evasion and enhances tumor cell reprogramming within the microenvironment [118,119].

In addition to its metabolic functions, PPARγ also plays a role in apoptosis and angiogenesis. All three PPARγ isoforms can increase the transcriptional activation of VEGF, a process that requires intermediary factors, and this activation is inhibited when MEK is blocked [120,121,122]. In BCa, metabolic reprogramming is a hallmark of the disease, and the overexpression of the lncRNA UCA1 promotes lipid accumulation by upregulating PPARα through miR-30a-3p. This dysregulated lipid metabolism prevents epirubicin-induced apoptosis, contributing to chemotherapy resistance via the miR-30a-3p/PPARα and p-AKT/p-GSK-3β/β-catenin signaling pathways [123].

**Figure 5:**
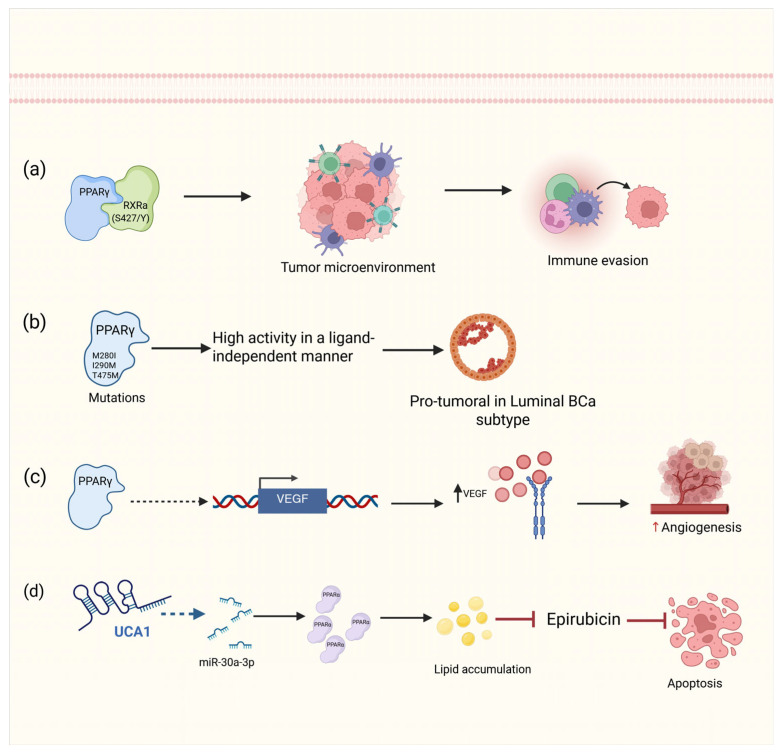
Roles of PPARγ and PPARα in BCa. (**a**) The formation of the PPARγ heterodimer with RxRa (S427/Y) regulates the tumor microenvironment by inhibiting the expression of inflammatory cytokines, leading to a state of immune evasion. (**b**) PPARγ point mutants transform cells into PPARγ-dependent cells by activating the pathway without the need for a ligand. (**c**) PPARγ transcriptionally activates the VEGF promoter via an indirect mechanism. (**d**) Through the microRNA miR30a-3p, the lncRNA UCA1 increases the expression of PPARα, which accumulates lipids that prevent epirubicin-induced apoptosis. Solid straight arrows indicate activation, dashed black and blue arrows indicate indirect activation, up black and red arrows indicate increased expression, red arrows represents inhibition process. Figure created with BioRender.com (accessed on 1 May 2025).

In summary, PPARs, particularly PPARγ, play dual roles in BCa depending on the tumor subtype and microenvironment. Its expression drives luminal differentiation but also supports tumor progression and immune evasion in basal subtypes. The complex interaction of PPARγ with other signaling pathways, such as those involving VEGF and the lncRNA UCA1, contributes to both metabolic reprogramming and chemotherapy resistance in BCa cells. Therefore, targeting PPARγ and its regulatory networks offers a potential therapeutic strategy to modulate tumor behavior and improve treatment efficacy (Figure 5).

## 7. Glucocorticoid Receptor Signaling in BCa: Isoform-Specific Roles and miRNA Interactions

GCRs play pivotal roles in mediating the effects of glucocorticoids, influencing various aspects of tumor growth and progression in BCa. GCR signaling, particularly through its isoforms, has complex roles in tumor biology. While GCR signaling can inhibit urothelial tumorigenesis through transrepression mechanisms, the GCR-β isoform has been shown to enhance BCa cell migration, suggesting its involvement in tumor invasiveness and metastasis [53,124]. This isoform-specific function points to a delicate balance in GCR activity, where its influence on BCa can vary depending on the context and the isoform involved.

Additionally, GCR signaling is involved in intricate crosstalk with other NRs, such as ERs, which further modulate BCa progression [125]. The interaction between GCRs and miRNAs highlights the complexity of their regulation in BCa. miRNAs can modulate GCR expression and activity, thus affecting tumor behavior and patient outcomes. Notably, miR-144 regulates GCRβ, increasing its expression, and promoting cell migration and metastasis through pathways such as the PI3K/AKT and NF-κB in vitro [126,127]. GCRβ, therefore, has been linked to increased cancer cell migration and EMT, key processes in tumor invasiveness [53,128]. These findings suggest that targeting either miR-144 or GCRβ could reduce tumor progression and metastasis.

**Figure 6:**
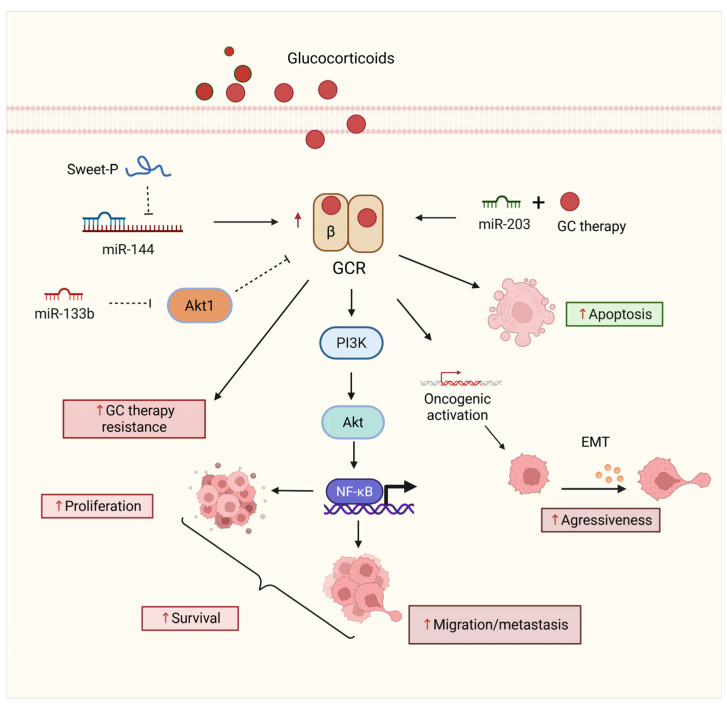
Regulation of GCR signaling by miRNAs in BCa progression and therapy response. The GCR axis plays a dual role in BCa, modulating survival, proliferation, and sensitivity to glucocorticoid therapy. miR-144 is repressed by the lncRNA Sweet-P, and miR-133b targets components of the PI3K/AKT pathway, contributing to therapy resistance and increased cell survival. Conversely, when coadministered with glucocorticoid therapy, miR-203 promotes apoptosis. GCR overexpression leads to oncogenic activation via NF-κB, enhancing migration, metastasis, and EMT. These interactions illustrate the potential of miRNAs to modulate glucocorticoid-based treatment efficacy and tumor aggressiveness. Solid straight black arrows indicate activation, dashed black arrow represents indirect inactivation, up red arrows indicate increased expression. Figure created with BioRender.com (accessed on 1 May 2025).

Moreover, miR-133b was identified as a regulator of GCR-related pathways, specifically targeting AKT1, which can contribute to glucocorticoid resistance [54,129]. These findings underscore the potential of combining miRNA modulation with GCR signaling to improve treatment efficacy. Restoring tumor-suppressive miRNAs or inhibiting oncogenic miRNAs may sensitize BCa cells to glucocorticoid treatment and other therapies. For example, the use of miR-203 mimics or enhancers alongside glucocorticoids has been shown to increase apoptosis in BCa cells, suggesting the therapeutic potential of combined miRNA and glucocorticoid treatment [130,131,132] (Figure 6).

In light of these insights, understanding the regulatory networks between miRNAs and GCR signaling presents exciting opportunities for novel therapeutic strategies. Targeting GCR signaling in combination with specific miRNA modulation could be an effective approach to improve BCa treatment outcomes. Additionally, miRNAs could serve as both biomarkers and therapeutic targets in BCa, aiding in the development of more personalized and effective treatment regimens. By integrating GCR and miRNA signaling into therapeutic frameworks, overcoming some of the current treatment limitations and enhancing the clinical management of advanced BCa may be possible.

## 8. NR-Targeted and Therapeutic Implications in BCa

NRs are critical targets in cancer therapy because of their central role in regulating cellular processes [133]. With the development of next-generation inhibitors, RNA-based therapeutics, selective modulators, and combination therapies, NRs have emerged as promising targets for therapeutic intervention in BCa. Among these AR antagonists, PPARγ agonists have demonstrated the potential to inhibit tumor growth by disrupting pathways involved in cellular proliferation, highlighting their therapeutic value in targeting BCa [113,134,135]. However, the clinical application of AR antagonists in BCa faces challenges related to resistance mechanisms and the heterogeneity of AR expression across BCa subtypes. This necessitates further refinement of AR-targeted therapies to improve their specificity and reduce potential resistance [136].

PPARγ activation has been linked to the regulation of inflammatory cytokines and immune responses in BCa, suggesting its potential as a therapeutic target. However, the role of PPARγ in BCa progression is dual; while it has tumor-suppressive effects in certain contexts, it also contributes to tumor aggressiveness in other contexts, depending on the tumor microenvironment. The activation of PPARγ in more aggressive BCa subtypes may enhance immune evasion and resistance to therapy, posing challenges for its therapeutic exploitation [119]. HNF4G, another key factor implicated in BCa progression, has emerged as another viable therapeutic target [136]. Targeting HNF4G could be particularly beneficial in cases where it drives metastatic and invasive behavior, although data on the effectiveness of targeting HNF4G remain limited and conflicting, particularly in the context of personalized medicine approaches.

Moreover, the activation of β-catenin by androgens underscores the potential of targeting AR signaling in BCa. While evidence suggests that inhibiting AR activity could block tumor progression, AR signaling also plays a key role in regulating BCa resistance to conventional therapies, making it necessary to combine AR-targeted treatments with other approaches, such as immune checkpoint inhibitors, to improve therapeutic outcomes [136].

### RNA-Based Therapeutics and Combination with miRNA-Based Therapies

RNA-based therapeutics, particularly noncoding RNAs, have emerged as promising strategies for BCa treatment. miRNAs, such as miR-34a, illustrate the potential of miRNA-based therapies for modulating NR activity and restoring tumor-suppressive miRNAs, as miR-34a inhibits tumor cell proliferation and invasion, suggesting that miRNA-based strategies could be used to regulate NR pathways and promote therapeutic outcomes in BCa [107]. While miRNA-based therapies show promise, their clinical implementation faces several challenges, particularly with miRNA delivery systems and ensuring that the modulation of these small molecules results in consistent therapeutic effects across BCa subtypes.

The knockdown of sex hormone receptors such as AR via siRNA has shown therapeutic potential by reducing BCa cell viability, providing further evidence that RNA-based strategies targeting NR signaling are involved in BCa progression [137]. However, resistance to siRNA therapies remains a critical limitation that must be addressed to fully realize the potential of this approach.

SERMs, such as raloxifene, have shown therapeutic potential by inhibiting urothelial carcinoma cell growth through ERβ-dependent mechanisms [138]. The challenge here lies in the selectivity of SERMs and their ability to target ERβ in a manner that avoids unwanted side effects associated with ERα activation. Moreover, selective modulation of ERβ expression has been linked to favorable prognostic outcomes and could further enhance therapeutic efficacy, although mixed results have been reported in clinical trials [90,139]. The interaction between estrogen signaling and BCa progression is particularly complex. ER signaling influences BCa progression and may enhance the efficacy of conventional therapies, suggesting that targeting ER signaling could also provide new avenues for treatment [94].

## 9. Combination Therapies and Personalized Approaches

Combination therapies that integrate NR modulators with other treatment approaches are gaining attention as promising strategies in BCa management. For example, combining MEK inhibitors with Bacillus Calmette–Guérin (BCG) therapy has shown enhanced efficacy in preclinical models of BCa [140]. Similarly, combining receptor tyrosine kinase (RTK) inhibitors with NR modulators may provide a synergistic effect, especially in tumors with high RTK expression [141]. While combination therapies show promise, the heterogeneity of BCa poses a significant challenge for the development of universal combination treatments. Tumors with varying levels of NR expression and activation may respond differently to the same combination, emphasizing the need for personalized therapeutic approaches.

The use of miRNAs in combination with NR-targeted therapies is another potential therapeutic strategy. For example, miR-124 enhances the efficacy of NR-targeted therapies by modulating key signaling pathways, such as the STAT3-NF-κB pathway, thereby reducing inflammation and angiogenesis [79,142]. miR-27b, which is overexpressed in BCa, has been shown to inhibit PPARγ and vitamin D receptor (VDR) in glioblastoma, reducing tumor progression [143]. miR-124 also regulates ONRs, promoting apoptosis and reducing cell proliferation in cancer and skeletal muscle [144], and could be another potential treatment target in BCa. These miRNAs could be used as part of a combination therapy strategy in BCa, alongside RNA-based therapies and SERMs/SARMs, creating a multifaceted approach to inhibit tumor growth and invasion.

Despite the promising potential of combining miRNA modulation with NR-targeted therapies, data on the effectiveness of such combinations remain inconsistent, particularly in clinical trials where the patient population is heterogeneous. These inconsistencies highlight the need for further research to identify biomarkers that can predict patient response to combination therapies, enabling the development of more tailored and effective treatments for BCa.

**Figure 7:**
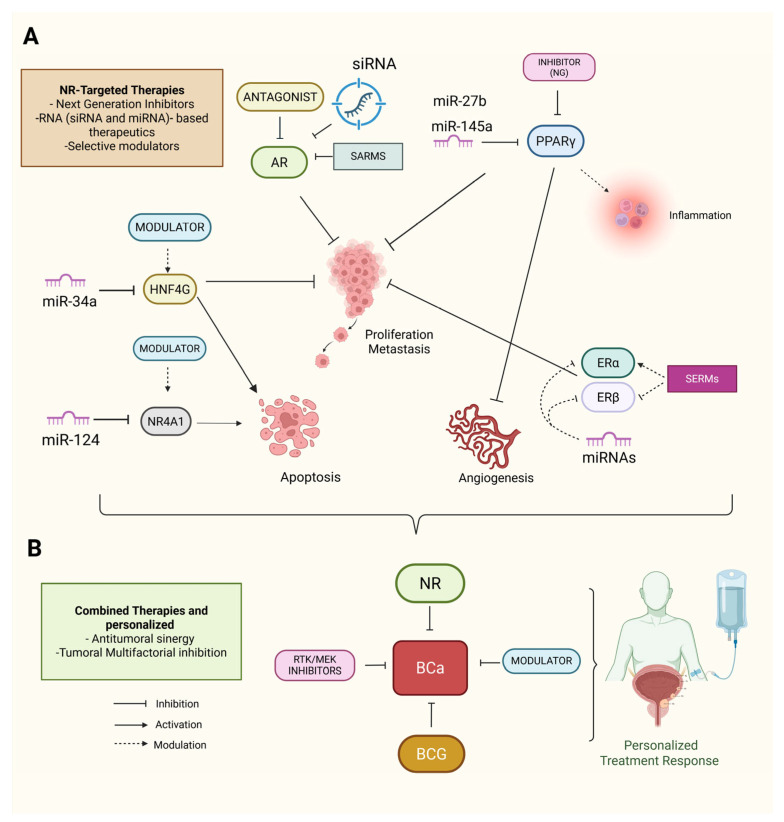
NR-targeted strategies and RNA-based therapeutics in BCa. (**A**) Schematic highlighting major therapeutic approaches directed at NRs implicated in BCa progression. AR antagonists (e.g., enzalutamide), PPARγ agonists, SERMs, SARMs, and next-generation NR inhibitors have demonstrated potential in modulating tumor cell proliferation and survival. RNA-based therapies, including miRNA mimics (e.g., miR-34a targeting HNF4G) and siRNA knockdown of AR, offer a complementary strategy involving interference with NR expression and activity. Additional miRNAs, such as miR-145, miR-124, and miR-27b, have been shown to modulate NR-related pathways, including PPARγ, VDR, and NR4A1 signaling, thus affecting inflammation, apoptosis, and therapeutic resistance. (**B**) Combining strategies integrating NRs and RNA-based tools may increase therapeutic efficacy and offer avenues for personalized BCa management. Solid straight black arrows indicate direct activation, curved dashed black arrows indicate indirect inhibition, dashed black arrows indicate indirect activation. Created with BioRender.com (accessed on 1 May 2025).

The interactions between miRNAs and NRs in BCa highlight the complexity of their regulatory roles in cancer progression. Combining miRNA modulation with NR-targeted therapies, such as RNA-based therapies, SERMs, and SARMs, presents a promising approach for personalized therapeutic strategies that can more effectively address tumor growth and invasion in BCa patients (Figure 7A,B). However, clinical trials are needed to validate the efficacy of these combinations and determine the best strategies for integrating them into treatment regimens.

## 10. Conclusions and Perspectives

The interplay between NRs and miRNAs plays a pivotal role in BCa biology by regulating key oncogenic processes such as cell proliferation, apoptosis, metastasis, and therapeutic resistance. This review highlights how miRNAs modulate NR expression and activity and, conversely, how NRs regulate miRNA biogenesis and functions, establishing complex regulatory circuits that drive tumor progression and treatment response. Specific NR–miRNA interactions, such as AR–miR-449a, ERβ–miR-92a, HNF4G–miR-34a, and PPARγ–miR-30a-3p, have been shown to influence tumor subtype specification, immune evasion, metabolic rewiring, and drug sensitivity. These molecular axes offer promising targets for therapeutic intervention. Furthermore, Nur77, RORC, and HNF4G are emerging as novel regulators in BCa and deserve further investigation. From a clinical perspective, identifying specific NR–miRNA signatures may aid in stratifying patients and predicting prognosis or therapeutic response. RNA-based therapeutics, including miRNA mimics or inhibitors and siRNA-mediated NR silencing, represent attractive strategies to disrupt these oncogenic networks. Future research should focus on validating these miRNA-NR interactions in large BCa cohorts and exploring their functional relevance in vivo.

Additionally, integrating transcriptomic, epigenetic, and single-cell analyses will be crucial for understanding the spatial and temporal dynamics of NR-miRNA regulation in the BCa TME. As molecular pathways involving NRs and miRNAs are further revealed, technologies such as CRISPR-Cas9 and advanced RNA sequencing could specifically target these molecular players. Combining NR-targeted therapies with immunotherapies or chemotherapies may improve treatment outcomes. Personalized medicine, which is based on genetic profiling of individual tumors, could become the gold standard for BCa treatment, ensuring that each patient receives the most effective therapy tailored to their tumor profile. These approaches could enhance treatment efficacy and reduce resistance by combining selective NR modulators (e.g., SERMs, SERDs, or other antagonists) or immune checkpoint inhibitors.

## Data Availability

The raw data supporting the conclusions of this article will be made available by the authors on request.

## Abbreviations

The following abbreviations are used in this manuscript:

BCa	Bladder cancer
NR	Nuclear receptor
BCG	Bacillus Calmette–Guérin
miRNAs	microRNAs
mRNAs	Messenger RNAs
PCa	Prostate cancer
MIBCa	Muscle-invasive BCa
NMIBCa	Non-muscle-invasive BCa
AR	Androgen receptor
AREs	Androgen response elements
UTR	Untranslated region
EMT	Epithelial-to-mesenchymal transition
ER	Estrogen receptors
PPARs	Peroxisome proliferator-activated receptors
ONRs	Orphan nuclear receptors
GCRs	Glucocorticoid receptors
VDR	Vitamin D Receptor
